# Effects of Feed Particle Size and Hydro-Thermal Processing Methods on Starch Modification, Nutrient Digestibility and the Performance and the Gastrointestinal Tract of Broilers

**DOI:** 10.3390/ani9060294

**Published:** 2019-05-30

**Authors:** Wendy Liermann, Mandy Bochnia, Andreas Berk, Verena Böschen, Liane Hüther, Annette Zeyner, Sven Dänicke

**Affiliations:** 1 Institute of Nutritional Physiology, Oskar Kellner, Leibniz Institute for Farm Animal Biology, 18196 Dummerstorf, Germany; 2 Institute of Animal Nutrition, Friedrich Loeffler Institut, Federal Institute for Animal Health, 38116 Brunswick, Germany; Andreas.Berk@fli.de (A.B.); Liane.Huether@fli.de (L.H.); Sven.Daenicke@fli.de (S.D.); 3 Group Animal Nutrition, Institute of Agricultural and Nutritional Sciences, Martin Luther University Halle-Wittenberg, 06120 Halle (Saale), Germany; mandy.bochnia@landw.uni-halle.de (M.B.); annette.zeyner@landw.uni-halle.de (A.Z.); 4 Research Institut of Feed Technology of the International Research Association of Feed Technology e.V., 38110 Brunswick, Germany; v.boeschen@iff-braunschweig.de

**Keywords:** broiler performance, technical feed treatment, proventriculus, gizzard

## Abstract

**Simple Summary:**

Technological benefits of feed processing methods are well defined but not their benefits and disadvantages for broiler feeding. The current study tested the impact of feed particle size and various hydro-thermal processing methods (HTPM) on feed value, broiler performance and alterations of the digestive tract. It was shown that HTPM influences physico-chemical characteristics of the feed including starch modifications. The compaction process during pelleting contributes to the reduction of feed wastage and selection. However, the high daily feed intake caused by pellet feeding is also a main risk factor for proventricular dilatation.

**Abstract:**

Influences of feed particle size (coarse, fine) and hydro-thermal processing methods (HTPM) (without–non-compacted feed, pelleting, expanding and pelleting) on feeding value and the performance and digestive tract of 624 broilers were studied. HTPM increased the starch disintegration of feed. Starch disintegration and electron microscopy indicated the highest degree of starch modification in expanded and pelleted feed. HTPM affected ether extract digestibility (*p* < 0.05). A grinding-by-HTPM interaction was found in case of crude protein digestibility (*p* = 0.008). Non-compacted feed reduced daily feed intake (DFI) and body weight gain and increased the feed to gain ratio compared to compacted feeds (*p <* 0.001). Compacted feeds increased proventricular size and the risk of *Isthmus gastrici* dilatation compared to coarsely ground non-compacted feed, except for finely ground expanded and pelleted feed. Finely ground feed reduced proventricular weights compared to coarsely ground feed and pelleted feed compared to other feeds. Non-compacted feed increased gizzard weights compared to compacted feeds. Relationships between proventricular size and *Isthmus gastrici* dilatation and the DFI were detected. Summarizing, the beneficial effects of pelleted feed were mainly based on the reduction of feed wastage and selection. However, the high DFI caused by pellet feeding is also a main risk factor for proventricular dilatation.

## 1. Introduction

Feed quality plays a key role in the successful production of livestock products. In this context, successful production means the exploitation of the performance potential of animals in consideration of animal health and welfare. In recent years, nutritional and physiological understanding promoted the development of new feed processing methods. Besides technological benefits, feed processing methods are associated with the reduction of feed wastage and feed selection by the animals, a decreased segregation of feed ingredients and reduction of pathogenic organisms as well as anti-nutritional factors [1,2]. Moreover, these methods result in physico-chemical changes of the feed which modify nutrient starch and proteins and can improve the palatability [1]. However, technically treated feeds are also known for adverse effects on the digestive tract and especially proventricular alterations in broilers [3]. Meanwhile, there is a wide range of methods which have to be compared with regard to their beneficial impact and the disadvantages from a nutritional point of view. Different experimental designs and feed components used in previous studies complicate a direct comparison of these methods. Therefore, the present examinations aimed to balance the beneficial effects and the disadvantages of various common technical feed treatments used in broiler feed production with regard to the impacts on the digestibility of feed, the broiler performance and the gastrointestinal development.

## 2. Materials and Methods

### 2.1. Experimental Diets and Design

The experiment included six feeding groups. The composition of the basal diet, which was formulated according to the requirements of growing broiler chickens [4], was similar for all treatment groups (Table 1 and Table 2).

Feeds differed on the one hand in particle size (coarse vs. fine) and on the other hand in hydro-thermal processing methods (HTPM), (without hydro-thermal treatment = non compacted feed, pelleted, expanded and pelleted) (Table 3). Therefore, a part of the mixed feed was milled completely by hammer mill (Tietjen Verfahrenstechnik GmbH, Hemdingen, Germany), either through a 6.0 mm screen (coarsely ground) or a 3.0 mm screen (finely ground). Then, the feeds were not further treated, solely pelleted or expanded and pelleted. Solely pelleted feeds were treated with a pellet-die press (Salmatec, Gödenstorf, Germany) at 75 °C and 3% steam supply. Expanded and pelleted feeds were expanded (expander from Amandus Kahl GmbH & Co. KG, Reinbek, Germany) at 130 °C and 3% steam and subsequently pelleted at 87 °C and 3% steam. Depending on throughput, the retention times in the expander take 5–8 s and in the pellet press 4–10 s. The steam supply was controlled with a flow measuring system type DVH-P (Heinrichs Messtechnik GmbH, Köln, Germany). The pellet diameter was 3 mm.

### 2.2. Growth Experiment

A total of 624 male broilers (Ross 308) were used for the feeding experiment which lasted 35 days. They were housed in 48 floor pens (pen size = 1.04 m^2^) (8 pens per feeding group) containing 13 broilers per pen which were integrated in a climate controlled building. The room temperature was decreased by 1 °C every second day from 36 °C to 19 °C on day 33 after hatching. On the first two days after hatching illumination period lasted 23 h and 16 h for the following days. The pens were littered with chopped straw. Dry feed and water were available ad libitum. However, pelleted feed was offered crumbled in the first week after hatching. The pens were equipped with bell drinkers (until 5 days after hatching) and nipple drinkers. On day eight after hatching broilers were vaccinated against Newcastle Disease Virus. Body weight (BW) and feed intake were recorded weekly per floor pen.

**Figure 1:**
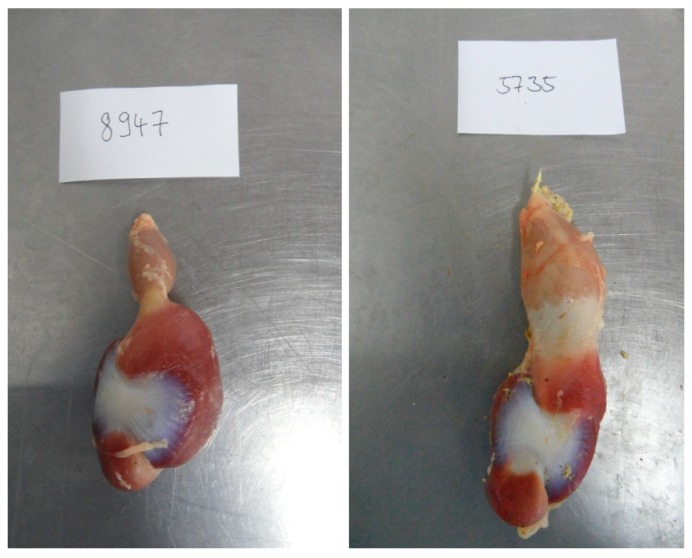
Example for assessment of the *Isthmus gastrici* (ISC), **left**: ISC = 0 (example animal fed coarsely ground non-compacted feed); **right**: ISC = 3 (example animal fed finely ground and pelleted feed)**.**

On day 35 after hatching 2 broilers per floor pen (16 broilers per group) which corresponded to the average live weight of the pen were slaughtered under conventional conditions. There was no fasting period before slaughtering. Chickens were electrically stunned, killed by bleeding, plucked and gutted. Thereafter, the head and the legs under the hock joint were cut and rejected. The remaining left leg, left breast meat, heart, liver without bile bladder, spleen, pancreas, abdominal fat and the remainder of the eviscerated body were weighed. The digestive tract was dissected cranial to the proventriculus until the cloaca. The different subsections of the digestive tract were separated (proventriculus, gizzard, duodenum, jejunum, ileum, caeca and rectum). The length of named digestive organs, except for the rectum, as well as the width of the proventriculus and the gizzard at the broadest part of the organs, was measured by calliper. The weights of the proventriculus, gizzard, duodenum, jejunum, ileum and caeca were determined which were emptied by cutting, flushing with water and subsequent wiping. A transversal section of the gizzard was prepared from its broadest extension to examine the thickness of the main muscles, *Musculi crassus caudodorsalis* and *cranioventralis*. Dilatation of the *Isthmus gastrici* was assessed by a scoring system (ISC, 0 = no obvious dilatation, 1 = moderate dilatation, 2 = middle degree of dilatation, 3 = high degree of dilatation, Figure 1).

Chyme samples of ileum were collected before this organ was rinsed with water. Therefore, the section between the Meckel’s diverticulum and the junction between the small intestine and caeca was separated by surgical clamps and scissors. Thereafter, one clamp was removed from the dissected part and the chyme was transferred in a 10 mL tube.

### 2.3. Balance Study

The balance study was in accordance with the guidelines by German animal protection law and approved by the Lower Saxony State Office for Consumer Protection and Food Safety, LAVES, Germany (registered under the number: 33.9-42502-04-082/09).

A total of 36 broilers (6 chicken per group; from 36 different pens) which corresponded to the average live weight of the group were separated from the growth experiment on day 14 after hatching for the balance trial. They were housed individually in balance cages without bedding and with visual contact to the other chicken. Balance cages were integrated in a climate controlled room. The temperature and light regimen corresponded to the growth experiment. Each cage was equipped with a feed trough and a water trough, which were installed on opposite sides of the cage. Broilers were given *ad libitum* access to feed and water.

The balance trial started after a seven day adaptation phase and included a six day sampling period. Broilers were weighed before starting the balance trial and at the end of the balance trial. Feed intake was also recorded during sampling period. Excreta were collected twice daily at 7:00 am and at 2:00 pm and pooled per chicken. Immediately after collection excreta were stored at −20 °C until further analyses.

Broilers were slaughtered by mechanical stunning and exsanguination on day 28 and 29 after hatching. The ileal chyme was collected as described for the growth experiment.

### 2.4. Analytical Methods

Calculations of particle size distribution of the coarsely ground non-compacted feed and the finely ground non-compacted feed was conducted before compaction by dry sieve analysis according to DIN 66165-1:1987-04 [5] and DIN 66165-2:1987-04 [6] using a sieve tower corresponding to DIN ISO 3310/1 [7].

Samples of feeds and excrements were prepared and analysed in accordance with the methods of the Association of German Agriculture Analysis and Research Centres (VDLUFA). Briefly, the methods of Naumann and Bassler [8] were used to investigate dry matter (DM) and additional crude nutrients. Nitrogen was determined by the combustion method according to Dumas [8]. The VDLUFA method 5.1.1 (HCL digestion) was used to analyse ether extract; 6.1.1 and 6.5.1 to analyse crude fibre and neutral detergent fibre (NDF) and 8.1 to analyse crude ash, respectively. Starch was measured enzymatically in feed and in ileal chyme (of chicken from balance study) using test kits from R-Biopharm AG (Mannheim, Germany). Starch disintegration in the feed was determined using the amyloglucosidase-method 7.2.6 of VDLUFA. Sugar was analysed according to Luff-Schoorl [8].

Apparent total digestive tract protein digestibility was determined as described by Pahle et al. [9], which required the analysis of the ninhydrin-positive α-amino-nitrogen in diets and excreta.

Furthermore, starch granules of feed and ileal chyme were visualized by electronic scanning microscopy (ESM) according to the method published by Bochnia et al. [10,11]. For ESM the feed samples were crushed (1 mm; Cyclotec Sample mill (Foss Tecator AB, Höganäs Sweden). A minimal percentage of the rough pulverized sample was obliterated on a metallic, round microscope slide with a diameter of nearly 1 cm, comparable to a knob battery. The samples were air dried and sputter coated with gold. Regarding the ileal chyme sample a special separation procedure was conducted. Briefly, 1/3 of each chyme sample was separated. Samples from broilers of the same feeding group were pooled. In order to verify that structures detected in ESM interpreted to be starch granules were indeed starch particles, the ileal chyme was first treated with Lugol’s solution (0.5 g iodine, 1 g potassium, 600 mL distilled water) and centrifuged at 3000 rpm for 10 min. The area of the sediment which was stained blue to purple was interpreted as the accumulated starch granules. This area was separated manually and the procedure was repeated until the starchy content seems to be largely separated. After the air-drying of this content, it was then investigated by ESM according to the procedure mentioned below for the feedstuffs.

A scanning electron microscope JSM 6300 (JEOL Ltd., Tokyo, Japan) was used, which works with a high-vacuum between 2.0 × 10^−2^ and 1.5 × 10^−6^. The electron beam is generated by stimulation a wolfram cathode. The commonly used excitation voltage is 20 keV, but starch granules ruptured, therefore it was adjusted only 15 keV. To realize the morphological analysis of the starch granules ESM photographs were used, which are so-called secondary electron pictures. For repeatability and to take representative pictures, a magnification of 200 was used to get a first general view followed by pictures in four different magnifications (× 500, 1000, 1500 and 3000) to take an optimal view on starch granules and their morphology.

Additionally, the feed extract viscosity and the viscosity of the ileal chyme were determined. Therefore, 1 g feed were mixed with 2 g distilled water. Feed-water mixture and ileal chyme were centrifuged for 15 min at 4 °C and 15,000 rpm. The resulting supernatant was divided into three aliquots (50 µL per aliquot) and transferred in a 2 mL Safe-Lock Tube. Thereafter, supernatant was centrifuged for 10 min at room temperature and 14,000 rpm (Centrifuge 5417R, Eppendorf, Hamburg, Germany). Subsequently, viscosity was measured by viscosimeter model DV-ii+ Viscometer (Brookfield Engineering Laboratories Vertriebs GmbH, Lorch, Germany) at a stable temperature of 40 °C and at 100 rpm.

### 2.5. Calculations and Statistical Analyses

The starch disintegration [%] of feed is based on the ratio of hydrolysed starch and crude starch multiplied by 100.

Performance parameters were calculated as followed:

Daily feed intake (DFI) [g] = feed consumption [g]/feeding period [d]/number of broilers per pen

BW gain (BWG) [g] = (final BW [g]-initial BW [g])/time of feeding period [d]/number of broilers per pen

Feed to gain ratio (FGR) [g/g] = DFI [g]/BWG [g]

Intestinal segment weight to length ratio (WL ratio) of the different intestinal sub-segments and the total small intestine was calculated as the quotient of the segment weight [g] and the segment length [cm].

For statistical analyses MIXED procedure of SAS Enterprise Guide 6.1 was used. The multifactorial variance analyses of nutrient digestibility, performance parameters, organ traits and ileal viscosity included the fixed effects “particle size” and “HTPM” as well as their interactions. Least squares mean (LsMeans) and standard errors (SE) were estimated for each fixed effect in the mentioned models and all differences of LsMeans were subsequently verified by the Tukey-Kramer test. The correlation coefficient according to Pearson was estimated using the SAS Enterprise Guide 6.1 and assessed as significant at a *p*-value < 0.05.

## 3. Results

Only one broiler fed coarsely ground non-compacted feed and one broiler fed finely ground and pelleted feed died during growth experiment. Furthermore, one broiler fed coarsely ground non-compacted feed was excluded from the balance trial because of anorexia.

### 3.1. Feed Analyses

Feed analyses of crude nutrients showed that feeds did not differ markedly in crude nutrient contents (Table 2). In general, slight differences were within the analytical latitudes. However, noticeably lower ether extract content were shown in solely pelleted feeds and coarsely ground, expanded and pelleted feed compared to other feeds. Furthermore, the latter feed showed slightly higher starch content compared to the other feeds.

Results of the particle size distribution of coarsely ground and finely ground feed before compaction are summarized in Table 4. Especially proportions of particles greater than 2000 µm differed markedly between coarsely ground and finely ground feed. While 40% of particles were greater than 2000 µm in coarsely ground feed the percentage of mentioned particles in finely ground feed amounted to only 6.8%.

Lowest feed extract viscosity was measured in finely ground non-compacted feed (Table 2). The highest feed extract viscosity was determined in coarsely ground and pelleted feed. Except for coarsely ground, expanded and pelleted feed hydro-thermally treated feeds showed higher extract viscosity than non-compacted feed with similar grinding extent.

**Figure 2:**
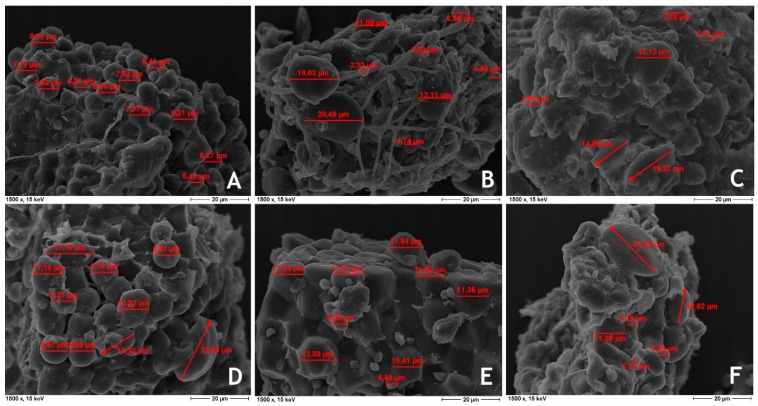
ESM picture from starch granules of broiler feed (× 1500) ((**A**) coarsely ground non-compacted; (**B**) coarsely ground and pelleted; (**C**) coarsely ground, expanded and pelleted; (**D**) finely ground non-compacted; (**E**) finely ground and pelleted; (**F**) finely ground, expanded and pelleted).

Non-compacted feed showed markedly lower starch disintegration than hydro-thermally treated feeds (Table 2). Expander treatment before pelleting resulted in a slight increase in starch disintegration compared to solely pelleted feed. Starch granules of coarsely ground (Figure 2A) and finely ground non-compacted feed (Figure 2D) had a spherical shape and were reliably separated. Visualized starch granules mostly originated from corn and wheat as the main starchy components in the basal diet showed equal dimensions (~10 µm) combined with strong and tight connections between each other comparable to mosaic-like fitting or bouldering. In contrast the spherical shape was lost after pelleting (Figure 2B,E) and expander treatment combined with pelleting (Figure 2C,F) and the granules seemed to swell and melt, whereas the differences between the coarsely and finely ground feeds over all treatments seems to be quite low. The most distinct changes of starch granules were shown in general after expander treatment combined with pelleting. The size of starch granules measured by ESM varied between 2.6 µm and 23.3 µm, whereas an increasing content of bigger granules (>10 µm) were observed in the hydro-thermal treated feeds (Figure 2B,C,E,F).

### 3.2. Digestibility of Crude Nutrients

Total digestive tract digestibility of organic matter and N-free extractives was not influenced by feed treatment (Table 5). HTPM tended to affect total digestive tract digestibility of crude fibre (*p* < 0.1). The highest crude fibre digestibility was determined in feeding groups fed pelleted feed. Finely ground non-compacted feed showed the lowest digestibility of crude fibre. Furthermore, HTPM significantly influenced the digestibility of ether extract (*p* < 0.05). Expanding and re-pelleting resulted in significantly higher total digestive tract ether extract digestibility compared to non-compacted feed (*p* = 0.046). There was a significant interaction between grinding and HTPM in case of crude protein digestibility (*p* = 0.008). While total digestive tract crude protein digestibility of pelleted feeds was higher compared to the total digestive tract digestibility of non-compacted feeds the total digestive tract crude protein digestibility of expanded and pelleted feeds was lower compared to non-compacted feeds.

**Figure 3:**
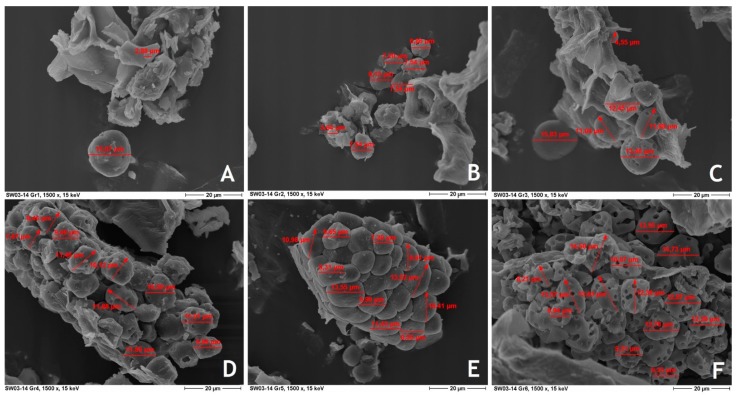
Electronic scanning microscopy (ESM) picture from starch granules of ileal broiler chyme (× 1500) ((**A**) coarsely ground non-compacted; (**B**) coarsely ground and pelleted; (**C**) coarsely ground, expanded and pelleted; (**D**) finely ground non-compacted; (**E**) finely ground and pelleted; (**F**) finely ground, expanded and pelleted).

To visualize the degree of starch digestion in ileal chyme ESM method was used. Figure 3 demonstrates that all visualized starch granules show pores/pits on the surface layer or to be completely permeated by pin holes which can be described by endocorrosion. There were also hints of exocorrosion (break down of starch over the entire surface of the granules), well known for highly digestible cereal grains. No direct relations to feed processing could be observed, however, starch granules of isolated from birds fed coarsely ground non-compacted feed and finely ground pelleted feed seemed to be less modified than starch granules from birds of other feeding groups. The starch granules of ileal chyme from broilers fed finely ground, expanded and pelleted feed showed the most pronounced modifications highlighted through a high content of pin holes. Starch content in the ileal chyme was not different between the feeding groups (*p* < 0.05).

### 3.3. Fattening and Slaughtering Performance

During growth experiment the highest DFI and the highest BWG were recorded after feeding pelleted feed. The lowest DFI and BWG were achieved by feeding non-compacted feed (Table 6). The differences between the mentioned feeding groups were significant (*p* < 0.001). Moreover, the BWG and DFI of broilers fed expanded and pelleted feed was significantly decreased compared to pelleted feed (*p* < 0.001), however, significantly increased compared to non-compacted feed (*p* < 0.001).

There was also a significant difference in DFI and BWG between broilers fed coarsely ground, expanded and pelleted feed and broilers fed finely ground, expanded and pelleted feed (*p* < 0.001). Furthermore, BWG of broilers fed coarsely ground and pelleted feed was significantly decreased compared to BWG of broilers fed finely ground and pelleted feed (*p* = 0.025). Non-compacted feed significantly increased the FGR compared to pelleted or expanded and pelleted feed (*p* < 0.001). A significant difference between FGR of feeding groups fed coarsely ground feeds and FGR of feeding groups fed finely ground feeds were not shown.

There was a significant grinding-by-hydro-thermal treatment interaction in case of BW measured on slaughtering day (*p* < 0.001). Feeding non-compacted feed significantly reduced the BW on slaughtering day compared to all other feeds (*p* < 0.001). In contrast, feeding of pelleted feed increased the BW of broilers compared to all other feeds (*p* < 0.001). Moreover, the BW of chicken fed finely ground and pelleted feed was significantly higher compared to chicken fed coarsely ground and pelleted feed (*p* < 0.001).

Grinding extent significantly influenced the weight of left leg and abdominal fat (*p* < 0.01) (Table 6). Generally, the pooled LsMeans of leg weight and abdominal fat were decreased by feeding finely ground feed compared to coarsely ground feed. The pooled LsMeans of breast meat weight were significantly lower in chicken fed non-compacted feed compared to chicken fed pelleted feed or expanded and pelleted feed (*p* < 0.001).

### 3.4. Organ Traits

Measured organ traits are summarized in Table 7.

There was a significant grinding-by-HTPM interaction in case of heart, liver and pancreas weights (*p* < 0.01). The weight of hearts of broilers fed finely ground, expanded and pelleted feed were significantly higher than heart weights of broilers fed non-compacted or finely ground and pelleted feed (*p* < 0.05). While highest liver weights were determined after feeding finely ground, expanded and pelleted feed or finely ground non-compacted feed lowest weights of this organ were observed in broilers fed coarsely ground non-compacted or finely ground and pelleted feed. Pancreas weights of broilers fed finely ground non-compacted feed were significantly higher than pancreas weights of broilers from other feeding groups (*p* < 0.01).

Grinding significantly influenced the relative weight of proventriculi (*p* = 0.029) but not the length and the width of this organ (*p* > 0.05). In contrast, HTPM significantly influenced all measured organ traits of proventriculus (*p* < 0.001). The pooled LsMeans of proventricular weights were significantly lower in chicken fed finely ground feed compared to chicken fed coarsely ground feed (*p* = 0.029). Moreover, pooled LsMeans of chicken fed non-compacted feed and expanded and pelleted feed were significantly higher compared to chicken fed pelleted feed (*p* < 0.001). Additionally, non-compacted feed resulted in a smaller proventriculus, indicated by lower length and width compared to other feeding groups, except for the proventriculi of broilers fed finely ground, expanded and pelleted feed, which showed a similar organ width. Broilers fed coarsely ground non-compacted or finely ground, expanded and pelleted feed showed lowest ISC. *Isthmus gastrici* of broilers fed finely ground non-compacted or finely ground and pelleted feed were classified with a moderate average ISC of 1.8 and 1.7, respectively. Coarsely ground and pelleted feed as well as coarsely ground, expanded and pelleted feed resulted in highest values of ISC. The ISC values of broilers fed coarsely ground non-compacted feed and finely ground expanded and pelleted feed differed significantly from ISC values of broilers fed coarsely ground and pelleted feed and coarsely ground expanded and pelleted feeds (*p* < 0.05).

In the case of gizzard weight, a significant interaction between grinding and HTPM was detected (*p* = 0.002). In general, non-compacted feed resulted in significantly higher gizzard weights than pelleted feed (*p* < 0.001). Furthermore, gizzard weights of broilers fed non-compacted feeds were significantly higher than gizzard weights of broilers fed coarsely ground, expanded and pelleted feed (*p* < 0.05). In contrast, broilers fed coarsely ground, expanded and pelleted feed showed significantly higher gizzard weights compared to broilers fed finely ground and pelleted feed (*p* = 0.005). The width of this organ was significantly influenced by both fixed effects (*p* < 0.01). A grinding-by-HTPM interaction was detected considering the length of gizzard (*p* = 0.033). Gizzards of broilers fed coarsely ground and pelleted feed were significantly longer than gizzards of all other feeding groups (*p* < 0.05) except for broilers fed coarsely ground expanded and pelleted feed. Pooled LsMeans of gizzard width were significantly lower in broilers fed finely ground feed compared to broilers fed coarsely ground and pelleted feed (*p* = 0.001). Moreover, the pooled LsMeans of broilers fed pelleted feed were significantly lower compared to broilers fed expanded and pelleted feed (*p* < 0.001). The sum of measured gizzard muscle thicknesses was not influenced by feeding different technically treated feeds.

A significant interaction between grinding and HTPM were found in case of the ratio between proventriculus and gizzard weights. This ratio of chicken fed coarsely ground non-compacted feed and chicken fed finely ground, expanded and pelleted feed were significantly higher compared to chicken fed coarsely ground, expanded and pelleted feed or chicken fed finely ground and solely pelleted feed.

A significant grinding-by-HTPM interaction was detected in case of the weight of the total small intestine in relation to the BW (*p* = 0.001). Total small intestinal weight of birds fed coarsely ground non-compacted was significantly increased compared to the weight of birds from other feeding groups (*p* < 0.01). Furthermore, weights of total small intestine dissected from broilers fed finely ground and pelleted feed were significantly reduced compared to intestinal weights of broilers fed finely ground non-compacted or expanded and pelleted feed. HTPM significantly influenced the weight of duodenum (*p* < 0.001). The pooled LsMeans of duodenal weights in chicken fed non-compacted feed were significantly higher compared to chicken fed the compacted feeds (*p* < 0.001). Significant grinding-by-HTPM interactions were found in case of jejunal and ileal weight (*p* < 0.01). Highest weights of these intestinal sub-segments were recorded in broilers fed coarsely ground and non-compacted feed which were significantly higher compared to broilers of other feeding groups except for jejunal weights of broilers fed non-compacted feed. Intestinal WL ratio of total small intestine and the WL ratio of its sub-segments were significantly influenced by both fixe effects. In general, the pooled LsMeans of the WL ratio of the total small intestine and of the sub-segments were higher in chicken fed coarsely ground feed compared to chicken fed finely ground feed (*p* < 0.05). The pooled LsMeans of the WL ratio of the total small intestine, the duodenum and the jejunum were significantly lower in broilers fed non-compacted feed compared to chicken fed compacted feeds (*p* < 0.05). Moreover, the pooled LsMeans of the WL ratio of the total small intestine and the jejunum were significantly higher in chicken fed pelleted feed compared to chicken fed expanded and pelleted feed. The pooled LsMeans of the ileal WL ratio in chicken fed non-compacted feed or expanded and pelleted feed were significantly lower compared to chicken fed pelleted feed (*p* < 0.001).

Ileal chyme viscosity of broilers fed finely ground, expanded and pelleted feed was significantly increased compared to viscosity of broilers fed other feed forms (*p* < 0.05).

## 4. Discussion

The present study aimed to assess the benefits and disadvantages of different common feed processing methods in broiler fattening. One focus of these examinations was to test the impact of these methods on feed with regard to starch modification and nutrient digestibility. Another focus lies on the impact of variously processed feeds on chicken performance and the gastrointestinal tract of broilers.

### 4.1. Impact on Starch Modification and Feed Digestibility

In contrast to the previous studies of Liermann et al. [12], only marginal effects of HTPM on starch disintegration of feed were detected with fattening pig feed in the present investigations despite similar processing conditions. Furthermore, only slight alteration could be visualized by ESM. A possible explanation could be the high content of plant oil in the present basal diet. As reported by Thomas et al. [13] the presence of lipids can impede the gelatinization of starch or enhance the gelatinization temperature of this polysaccharide. HTPM loosened the tight connections of starch granules, whereas expander treatment seemed to be most effective.

Starch granules of ileal chyme showed characteristic pores and pin holes, which are clear indications of enzymatic attack of amylase as previously demonstrated by Fuwa et al. [14] and Kienzle et al. [15]. Slightly visual differences were observed between chickens fed coarsely ground and pelleted feed. Starch granules found in ileal chyme of chickens fed finely ground and pelleted feed were in a high extent attacked by digestive enzymes. The number and size of pin holes increase with increasing small intestinal starch digestibility [15,16]. Small pits can be interpreted as precursors of pin holes [15]. It has to be emphasized that the slightly lower modification of starch in ileal chyme of broilers fed coarsely ground non-compacted and finely ground and pelleted feed might be less an indication for lower digestibility of the whole dietary starch but rather than an indication for the low digestibility of these visualized starch granules themselves. Already, Fuwa et al. [14] demonstrated by ESM that the granules of some plants that are resistant to the action of amylases show surfaces similar to intact granules after enzyme attack. Moreover, it is assumed that only less digestible starch granules reach the chosen location of chyme collection. Interestingly, no indications of starch modification by HTPM as demonstrated in Figure 1 were shown in starch granules of ileal chyme of all chicken. This aspect could be an evidence for the rapid digestion of modified starch. The aspect that the starch content in ileum did not differ markedly between feeding groups may indicate that there were no differences in starch digestibility between these groups.

Although the protein digestibility was affected in a HTPM and grinding depended manner coarsely ground non-compacted feed showed no lesser protein digestibility compared to the other feeds indicating no beneficial effects of a higher processing degree on these parameter.

Coarsely ground feed was not lesser digestible than finely ground feed which might be due to the pronounced capability to grind coarse chyme-components by the gizzard in the presence of sufficient grit [17].

Pelleting resulted in slightly higher total digestive tract crude protein digestibility compared to expanded and pelleted feed. It is known that high temperatures during expander treatment can result in denaturation of proteins or destruction of feed additives such as enzymes which promote digestibility. Furthermore, interactions of high temperatures, moisture and sugar could result in the development of indigestible Maillard products [13,18,19]. These adverse effects on feed could also be responsible for the lower protein digestibility of expanded and pelleted feed compared to solely pelleted feed. The improvement of total digestive tract crude fibre and ether extract digestibility compared to non-compacted feed caused by HTPM might be a result of higher cell wall disruption and the release of integrated fat of feed components [20].

It is known that there are strong relations between nutrient digestibility or chicken performance and chyme viscosity [21,22]. Furthermore, previous studies demonstrated an impact of technical feed treatment on chyme viscosity [23,24]. In the present study, no effect on chyme viscosity was expected to arise from non-starch polysaccharides (NSP) by technical feed treatment because of the addition of NSP-hydrolysing enzyme (xylanase, β-glucanase) to the feed. As reported in the studies of Salih et al. [21]; Inborr and Bedford [23] and Dänicke et al. [25], chyme viscosity can be markedly reduced by supplementation of the mentioned enzymes. Indeed, chyme viscosity was low in all chickens. Nevertheless, finely ground expanded and pelleted feed resulted in significantly higher viscosity of ileal chyme compared to all other feeds. Interestingly, the highest chyme viscosity of broilers fed finely ground, expanded and pelleted feed was not related with the highest feed extract viscosity. Possibly, the increase in ileal chyme viscosity is related to the damage of supplemented enzymes by the high processing temperatures. In studies by Inborr and Bedford [23] and Vranjes et al. [19], markedly decreased enzyme activities were shown due to pelleting or extrusion. However, this effect was not observed when feeding coarsely ground, pelleted and expanded feed although equivalent processing temperatures were used. Because of the small differences of ileal chyme viscosity between feeding groups no obvious effects on nutrient digestibility were detected due to different technically treated feeds.

### 4.2. Impact on Fattening and Slaughtering Performance

Broilers fed non-compacted feed were markedly inferior compared to broilers fed compacted feeds considering BWG which in turn reduced the BW on slaughtering day. The lower BW of the broilers fed non-compacted feed resulted on the one hand in lighter left breast meat and on the other hand in a lower weight of abdominal fat. Decreased BWG was clearly caused by the lower DFI of non-compacted feed fed broilers. This result seemed to be mainly based on feed form which inhibited the ability of broilers to consume similar quantities of feed by the beak as compared to their compacted-feed fed counterparts. In case of the coarsely ground non-compacted feed also a selective feed intake can be considered as total feed intake-decreasing factor. This inhibition of the ability to consume the feed due to feed form resulted in turn in a higher feed wastage and spillage by the birds which explained the increased FGR of birds fed non-compacted feeds although no differences in feed digestibility compared to compacted feed were observed.

Expanded and pelleted feed reduced also the BWG compared to pelleted feed. Although the feed form did not differ from solely pelleted feed, also in this case the reduction of the DFI is the main reason for this decreasing effect. Possibly, this effect is based on differences in pellet texture and quality. Indeed, it is known that expanding influences pellet hardness and durability inter alia by starch modification [13], as also observed by ESM which plays a key role in the pellet quality and texture [26,27]. That these characteristics can affect broiler performances was proven by Parsons et al. [28]. Also, the differences in broiler performance between coarsely ground and finely ground expanded feeds might be explained by effects on pellet quality and texture. Interestingly, a fine grinding seemed to have adverse effects on the fattening performance when the feed was additionally expanded and pelleted compared to coarsely ground expanded and pelleted feed. In contrast, a fine grinding slightly increased the fattening performance when the feed was fed in meal form or pelleted compared to their coarsely ground counterparts. Possibly, these effects are also based on differences in feed texture and pellet quality which affected the DFI. On the other hand, it could also be a result of the differences in the protein digestibility of expander treated feeds, which was slightly reduced in finely ground expanded and pelleted feed compared to coarsely ground and pelleted feed. Furthermore, the starch content was slightly higher in coarsely ground expanded and pelleted feed compared to finely ground expanded and pelleted feed which might be also an influencing factor on BWG. Also, in the case of birds fed expanded and pelleted feed, the lower fattening performance compared with birds fed pelleted feed resulted in a slightly lower slaughtering performance.

### 4.3. Impact on Organ Traits

Chicken fed pelleted feed showed increases in proventriculus size but decreases in organ weight compared to other feeding groups. Therefore, it is more likely that the organ was stretched than an increase in muscular tissue occurred. It is suggested that a massive expansion of the proventriculus will limit the available space for other visceral organs in the abdominal cavity. The authors Jones and Taylor [29] and Taylor and Jones [30] derived a relationship between proventricular dilatation and the incidence of ascites.

There was a significant positive correlation between the DFI of broilers considering the whole fattening period and the ISC (*r* = 0.307; *p* = 0.002) as well as the width (*r* = 0.401; *p* < 0.001) and length (*r* = 0.404; *p* < 0.001) of the proventriculus. While the width and the length of the proventriculus were positively correlated to the DFI the weight of this organ was negatively correlated to the DFI (*r* = −0.544; *p* < 0.001). Therefore, a higher DFI caused a lighter but greater proventriculus. These aspects clearly indicate that the high DFI of chicken fed pelleted feed directly increase the risk for the development of proventricular dilatations in broilers. Furthermore, it is the explanation for the lower ISC and proventricular width of broilers fed coarsely ground non-compacted feed which showed lower DFI than broilers of other groups. Already Svihus and Hetland [31] have reported that some chickens tend to overconsume when feeding on finely ground, pelleted feed. Svihus et al. [32] recommended the inclusion of whole wheat in the diet to avoid overconsumption. However, a high DFI is not the explanation for the low ISC of broilers fed finely ground, expanded and pelleted feed because these broilers consumed similar amounts of feed compared to broilers of the other feeding groups which had higher ISC. Possibly, there is a relationship to the higher ileal chyme viscosity measured in the intestine of these broilers.

In contrast to the studies of Jones and Taylor [29], which included whole grains in pellets, coarsely grinding before pelleting could not inhibit the dilatation of the proventriculus, although grinding appears to affect the proventricular weight. Possibly, the differences in particle size between coarsely ground and pelleted feed as well as finely ground and pelleted feed were balanced after the compacting process by secondary grinding which was described in previous studies of Wolf et al. [33].

Compacted feed significantly decreased the gizzard weight compared to coarsely ground non-compacted feed. The heaviest gizzards had broilers fed coarsely ground non-compacted feed. Similar results were reported by Attia et al. [34] and Preston et al. [24]. The latter authors associated an increase of gizzard weight with more developed gizzard muscles. In contrast to this suggestion, no significant differences between the thicknesses of gizzard muscles of the different feeding groups were proven in the present study. Furthermore, no significant correlation existed between gizzard weight and thickness of main muscles (*r* = 0.037; *p* = 0.720). In studies of Wu et al. [35] the increase in gizzard weight was also not related to a thicker *tunica muscularis*. A general increase of this organ in broilers fed coarsely ground non-compacted feed compared to broilers fed other feeds, as assumed by Betscher et al. [3], was not confirmed. In the present study only the thick, lateral main muscles were considered. Perhaps, higher gizzard weights are based on alterations in the development of the thin craniodorsal and caudoventral muscles.

Svihus et al. [36] and Svihus [32] hypothesized that structural feed supports the development of a heavier gizzard and gizzard functionality. These authors also suggested that this effect improved the ability of the gizzard to regulate the feed intake and in turn avoid overconsumption. Indeed, the weight of the gizzard was negatively correlated to the DFI (*r* = −0.678; *p* < 0.001) in the present study. Furthermore, a significant negative correlation between gizzard weights and the BWG was detected (*r* = −0.575; *p* < 0.001).

In general, it should be noted that the minimum recommended particle sizes (30% particles > 1000 mm) by Svihus [32] were fulfilled in both non-compacted feeds. However, it has to be emphasized that a secondary grinding by compacting processes might reduce feed particle size of these feed variants. Therefore, it is assumed that the structural requirements of broilers fed hydro-thermal processed feed were unmet. During slaughtering of broilers from growth experiment often bedding material was detected in the gastrointestinal tract. Apparently, chicken fed compacted feed seemed to consume more litter material than chicken fed non-compacted feed. Svihus [32] discussed that the intake of litter material is the consequence of the compensation-demand of poultry after feeding poorly structured feed.

In accordance to studies of Taylor and Jones [30] who reported correlations between proventricular dilatation and duodenal WL ratio significant correlations were detected between duodenal WL ratio and proventricular size (proventricular length: *r* = 0.414, *p* < 0.001; proventricular width: *r* = 0.254, *p* = 0.013). Also, the WL ratio of further intestinal segments and of the total small intestine was correlated with proventricular length and width (*r* > 0.28; *p* < 0.01). These data clearly indicate that alterations in the upstream digestive tract also changes morphometric characteristics in the downstream digestive tract. Possibly, these effects are based on physico-chemical changes in the intestinal content. However, the intestinal WL ratio did not correlate to chyme viscosity in the present study as previously reported by Taylor and Jones [30]. Further, characteristics of the intestinal chyme which are known to differ after feeding different technically treated feeds are for example the particle size distribution or the pH [3,37,38].

Significant treatment-associated differences between weights of heart, liver and pancreas are inconsistent and neither explainable by higher protein level of feeds nor by higher digestibility of feed or performance. Betscher et al. [3] demonstrated in a meta-study that the inclusion of whole wheat results in a significantly increased pancreas weight. They associated the higher organ weight with an increase in pancreatic secretion. In the present study, finely ground non-compacted feed significantly increased the weight of the pancreas compared to all other feeding groups. As reported in studies of Nir et al. [39] overfeeding resulted in higher weight of pancreas and liver, however not in higher heart weight. Moreover, these organ weights did not correspond with the DFI measured in the present study.

## 5. Conclusions

Compacting processes are important to avoid feed wastage and feed selection. However, at the same time the high feed intake and a possible overconsumption due to feeding pelleted feed will markedly increase the risk of proventricular dilatation. It has to be emphasized that both the avoidance of feed wastage and the animal health are critical factors of a resource conserving production of livestock products.

## Figures and Tables

**Table 1:** Ingredients of the basal diet.

Ingredients	g/kg Feed
Corn	240.0
Wheat	353.8
Wheat meal	30.0
Soybean meal	250.0
Rapeseed expeller	60.0
Plant oil	35.0
Calcium carbonate	12.4
Calcium sodium phosphate	3.8
Calcium hydrogen phosphate	3.0
Premix ^†^	12.0
Calculated composition	
Metabolizable energy_,_ ME MJ/kg feed	13.0
Crude protein, g/kg feed	205
Crude fat, g/kg feed	70
Crude fibre, g/kg feed	31
Crude ash, g/kg feed	49
Lysine, g/kg	11.8
Methionine, g/kg	5.5
Calcium, g/kg feed	8.5
Phosphorus, g/kg feed	5.5

^†^ delivers per kg feed: 12,000 IU vitamin A; 4400 IU vitamin D_3_; 60 mg vitamin E; 60 mg iron; 11.3 mg copper; 67.5 mg zinc; 75 mg manganese; 1.5 mg iodine; 0.23 mg selenium; 1220 U xylanase; 152 U glucanase; 750 U phytase.

**Table 2:** Analysed crude nutrients (on dry matter basis).

Feeding Group	Grinding	Hydro-Thermal Processing Methods	Dry Matter [%]	Crude Protein [g/kg]	Ether Extract [g/kg]	Crude Fibre [g/kg]	NFE [g/kg]	NDF [g/kg]	Crude Ash [g/kg]	Starch [g/kg]	Sugar [g/kg]	Starch Disintegration [%]	Feed Extract Viscosity [mPas]
1	Coarsely ground	Non-compacted	90.8	229	88.1	34.9	597	125	50.8	426	48.8	6.5	1.22
2		Pelleted	87.9	237	72.8	33.3	612	129	44.7	466	51.2	11.5	1.38
3		Ex+P	89.8	208	76.4	35.9	642	139	37.3	492	49.9	12.3	1.16
4	Finely ground	Non-compacted	90.3	234	87.2	33.5	593	131	53.0	420	49.6	5.5	1.12
5		Pelleted	87.8	250	79.1	36.0	589	130	45.8	429	54.2	8.7	1.19
6		Ex+P	90.4	243	85.9	32.1	590	147	48.5	413	55.4	11.7	1.31

Ex + P = expanded and pelleted. NFE = N-free extractives. NDF = neutral detergent fibre.

**Table 3:** Experimental design: Combination of technical feed treatments.

Feeding Group	Grinding		Hydro-Thermal Processing Methods
	Particle Size	Screen Size of Sieves	
1	Coarse	6.0 mm	Without (non-compacted)
2	Coarse	6.0 mm	Pelleted
3	Coarse	6.0 mm	Expanded and pelleted
4	Fine	3.0 mm	Without (non-compacted)
5	Fine	3.0 mm	Pelleted
6	Fine	3.0 mm	Expanded and pelleted

**Table 4:** Particle size distribution of coarsely and finely ground non-compacted before compaction.

Particle Size	Particle Size Distribution [%]	
	Coarsely Ground	Finely Ground
<125 µm	2.0	2.3
125–355 µm	11.2	15.7
360–1000 µm	18.0	30.0
1005–2000 µm	28.9	45.2
>2000 µm	40.0	6.8
D50 [mm]	1.65	1.04

D50 = cumulative particle size distribution at 50%.

**Table 5:** Total digestive-tract apparent digestibility [%] of selected crude nutrients according to feeding groups examined in balance trials (LsMeans, *n* = 6).

Variable	Coarsely Ground			Finely Ground			SE	*p*-Value		
	M	P	Ex + P	M	P	Ex + P		Grinding	HTPM	Grinding × HTPM
Organic matter	77.0	77.7	75.8	74.8	76.8	75.5	1.47	0.343	0.512	0.805
CP digestibility	94.0 ^ab^	94.1 ^ab^	92.3 ^bc^	93.1 ^bc^	96.4 ^a^	90.8 ^c^	0.63	0.951	<0.001	0.008
Ether extract	88.8	90.2	90.4	87.9	89.7	90.6	0.86	0.569	0.048	0.842
Crude fibre	10.7	22.0	17.0	9.5	18.4	12.3	4.20	0.360	0.073	0.918
NFE	83.0	82.5	80.4	82.4	81.1	80.7	1.25	0.580	0.256	0.810

SE = Standard error. M = Non-compacted. P = Pelleted. Ex + P = Expanded and pelleted. HTPM = Hydro-thermal processing methods. CP = Crude protein. NFE = N-free extractives. ^a,b,c^ Different subscripts mark significant differences between feeding groups.

**Table 6:** Fattening and slaughtering performance and water consumption (LsMeans).

Variable	Coarsely Ground			Finely Ground			SE	*p*-Value		
	M	P	Ex + P	M	P	Ex + P		Grinding	HTPM	Grinding × HTPM
Fattening performance (*n* = 8), 0–35 day old broilers										
Daily feed intake [g/chicken]	60.8 ^d^	95.1 ^a^	83.1 ^b^	63.4 ^d^	97.2 ^a^	76.9 ^c^	1.2	0.621	<0.001	0.001
Body weight gain [g/chicken/day]	38.7 ^e^	64.8 ^b^	58.2 ^c^	40.9 ^e^	69.0 ^a^	54.3 ^d^	0.9	0.254	<0.001	<0.001
Feed to gain ratio [g/g]	1.66 ^a^	1.46 ^b^	1.43 ^b^	1.70 ^a^	1.38 ^b^	1.46 ^b^	0.02	0.770	<0.001	0.008
Slaughtering performance (*n* = 16), 35 day old broilers										
Body weight [kg]	1.5 ^d^	2.3 ^b^	2.2 ^c^	1.5 ^d^	2.5 ^a^	1.9 ^c^	0.03	0.361	<0.001	<0.001
Left breast meat [g/kg BW]	76.4	87.9	87.5	78.9	90.8	86.0	2.1	0.449	<0.001	0.500
Left leg [g/kg BW]	101.2	101.2	100.3	96.6	98.8	97.9	1.1	0.001	0.583	0.494
Abdominal fat [g/kg BW]	12.4	14.9	14.2	10.1	13.7	12.5	0.6	<0.001	<0.001	0.657

SE = Standard error. M = Non-compacted. P = Pelleted. Ex + P = Expanded and pelleted. HTPM = Hydro-thermal processing methods. ^a,b,c,d^ Different subscripts mark significant differences between feeding groups.

**Table 7:** Organ traits (LSMeans; *n* = 16), 35 day old broilers.

Variable	Coarsely Ground			Finely Ground			SE	*p*-Value		
	M	P	Ex + P	M	P	Ex + P		Grinding	HTPM	Grinding × HTPM
Organ weight [g/kg body weight]										
Heart	4.8 ^b^	4.9 ^ab^	4.9 ^ab^	4.6 ^b^	4.5 ^b^	5.3 ^a^	0.1	0.690	<0.001	0.006
Liver	20.9	21.7	22.8	22.4	20.2	21.6	0.5	0.287	0.032	0.002
Proventriculus	4.5	3.6	4.4	4.4	3.4	4.0	0.1	0.029	<0.001	0.711
Gizzard	17.4 ^a^	11.7 ^cd^	12.6 ^c^	15.6 ^ab^	9.2 ^d^	14.3 ^bc^	0.6	0.099	<0.001	0.002
ISC	0.9 ^b^	2.2 ^a^	2.0 ^a^	1.8 ^ab^	1.7 ^ab^	1.0 ^b^	0.2	0.179	0.014	<0.001
Pancreas	2.3 ^b^	2.3 ^b^	2.3 ^b^	2.9 ^a^	2.0 ^b^	2.2 ^b^	0.1	0.701	<0.001	<0.001
Total small intestine	32.0 ^a^	26.3 ^bc^	26.0 ^bc^	28.5 ^b^	24.0 ^c^	27.3 ^b^	0.6	0.005	<0.001	0.001
Duodenum	6.0	4.9	5.5	6.0	4.6	5.1	0.2	0.146	<0.001	0.629
Jejunum	13.3 ^a^	11.5 ^bc^	10.9 ^bc^	12.1 ^ab^	10.4 ^c^	11.6 ^bc^	0.3	0.070	<0.001	0.008
Ileum	12.7 ^a^	10.0 ^bc^	9.6 ^bc^	10.5 ^b^	9.0 ^c^	10.5 ^b^	0.3	0.005	<0.001	<0.001
Ceaca	4.5	2.9	2.9	3.3	8.1	5.0	2.2	0.270	0.716	0.362
Section extent [cm]										
Proventriculus length	2.97	3.29	3.36	2.91	3.30	3.22	0.08	0.336	<0.001	0.623
Proventriculus width	1.84	2.11	2.09	1.88	2.09	1.88	0.06	0.206	<0.001	0.076
Gizzard length	5.51 ^b^	5.98 ^a^	5.63 ^ab^	5.45 ^b^	5.42 ^b^	5.52 ^b^	0.08	0.005	0.107	0.033
Gizzard width	4.33	4.19	4.39	4.04	3.87	4.28	0.09	0.001	0.003	0.380
Gizzard muscles	3.40	3.11	3.18	3.31	3.20	3.18	0.11	0.967	0.154	0.709
Intestinal segment weight to length ratio [g/cm]										
Duodenum	0.31	0.36	0.37	0.31	0.36	0.33	0.01	0.038	<0.001	0.121
Jejunum	0.27	0.31	0.28	0.24	0.30	0.26	0.01	0.001	<0.001	0.564
Ileum	0.24	0.27	0.24	0.19	0.24	0.22	0.01	<0.001	<0.001	0.138
Total small intestine	0.26	0.30	0.28	0.23	0.28	0.25	0.01	<0.001	<0.001	0.363
Ileal chyme viscosity [mPas]										
Viscosity	1.86 ^a^	1.84 ^a^	1.74 ^a^	1.71 ^a^	1.61 ^a^	2.24 ^b^	0.07	0.487	0.001	<0.001

SE = Standard error. M = Non-compacted. P = Pelleted. Ex+P = Expanded and pelleted. HTPM = Hydro-thermal processing methods. ISC = Isthmus score. Gizzard muscles = Sum of thickness of *musculus crassus caudodorsalis* [cm] and thickness of *musculus crassus cranioventralis* [cm]. ^a,b,c,d^ Different subscripts mark significant differences between feeding groups.
